# Emphysematous osteomyelitis of the spine: A rare case report

**DOI:** 10.1097/MD.0000000000021113

**Published:** 2020-07-10

**Authors:** Sahyun Sung, Byung Ho Lee, Jung-Hwan Kim, Yung Park, Joong Won Ha, Seong-Hwan Moon, Hwan-Mo Lee, Ji-Won Kwon

**Affiliations:** a Department of Orthopedic Surgery, Yonsei University College of Medicine; b Department of Orthopedic Surgery, College of Medicine, Ewha Womans University Seoul Hospital, Seoul; c Department of Orthopedic Surgery, National Health Insurance Service Ilsan Hospital, Goyang, Korea.

**Keywords:** computed tomography, emphysematous osteomyelitis, *Escherichia coli*, spine, surgery

## Abstract

**Rationale:**

Emphysematous osteomyelitis is a rare disease caused by gas-forming bacteria. But only 45 cases have been reported in the literature since then.

**Patient concerns:**

A 72-year-old female presented to our hospital with severe lower back pain that aggravated 4 days ago.

**Diagnoses:**

Computed tomography (CT) revealed intraosseous mottled air in the T12 and L1 vertebral bodies and epidural space. The enhanced T1 and T2 magnetic resonance imaging scans showed heterogeneous signal intensity of vertebral bodies, suggestive of emphysematous osteomyelitis.

**Interventions:**

Surgery was performed to identify culture strains and to remove emphysematous lesions of the vertebral body using extensive transpedicular irrigation.

**Outcomes:**

*Escherichia coli (E coli)* was identified in the surgical specimen, and intravenous antibiotic therapy was continued with cefotaxime. The patient had a significant decrease in lower back pain after the surgery and the final CT scan before discharge revealed significantly decreased air at T12 and L1 vertebral bodies and no air density in the epidural space.

**Lessons:**

We present a patient diagnosed with emphysematous osteomyelitis in vertebral bodies caused by *E coli* and successfully treated with surgical intervention.

## 1 Introduction

Emphysematous osteomyelitis is a rare disease caused by gas-forming bacteria. It was first described in 1981, and only 45 cases have been reported in the literature since then.^[1]^ In extra-axial skeletons, most cases can be diagnosed by confirming intraosseous gas through computed tomography (CT).^[2,3]^ However, intraosseous gas in the vertebral body is more likely to be caused by noninfectious conditions such as degenerative disease, osteonecrosis, and neoplasm rather than emphysematous osteomyelitis; therefore, thorough differential diagnosis is necessary.^[4–6]^ We present a patient diagnosed with emphysematous osteomyelitis in vertebral bodies caused by *Escherichia coli (E coli)* and successfully treated with surgical intervention.

## 2 Case presentation

A 72-year-old female presented to the Emergency Department with severe lower back pain. She was on medication for hypertension and diabetes mellitus (DM) type 2. The patient had suffered from chronic lower back pain for 5 years, and was being treated with steroid injections at a local clinic periodically. The most recent injection was administered 4 days ago and she was referred to department of orthopedics due to aggravating back pain and thrombocytopenia, with a platelet count of <8000.

Her initial laboratory results showed neutrophilic leukocytosis (WBC count 11,670 cells/μL, segmented neutrophil 93.8%) and thrombocytopenia (platelet count 5000cells/μL). The erythrocyte sedimentation rate was 51 mm/h (0–20 mm/h) and C-reactive protein (CRP) was 9.88 mg/dL (0.00–1.00 mg/dL).

Her vital signs were as follows: temperature 37.6°C, pulse rate 95 bpm, and blood pressure 132/68 mmHg. Initial oxyhemoglobin saturation (SpO_2_) was 87% by pulse oximetry and SpO_2_ improved to 95% after applying 4 L of oxygen by mask.

**Figure 1:**
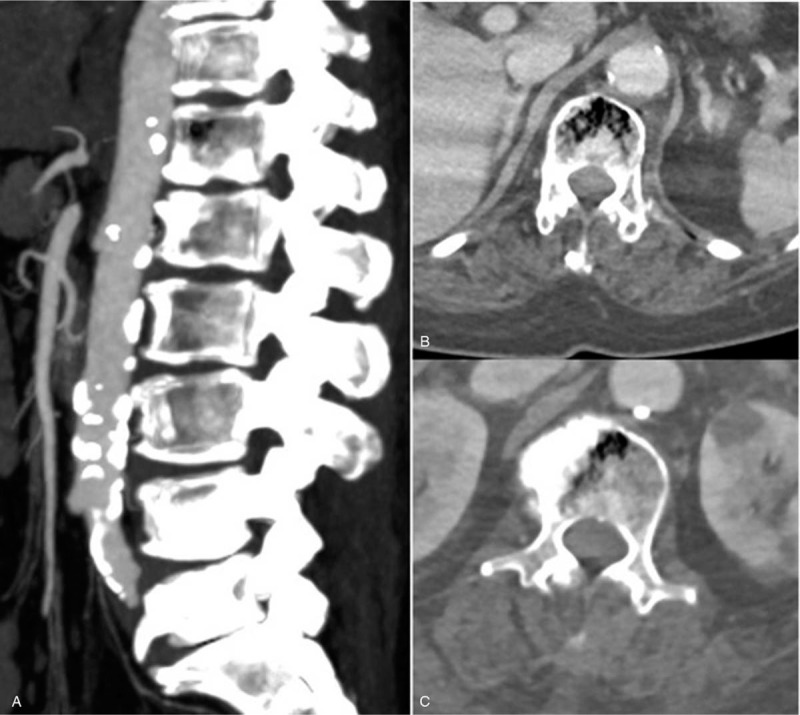
Chest and abdominal computed tomography scan at the time of admission showing intraosseous mottled air in the T12 (B) and L1 (C) vertebral bodies.

**Figure 2:**
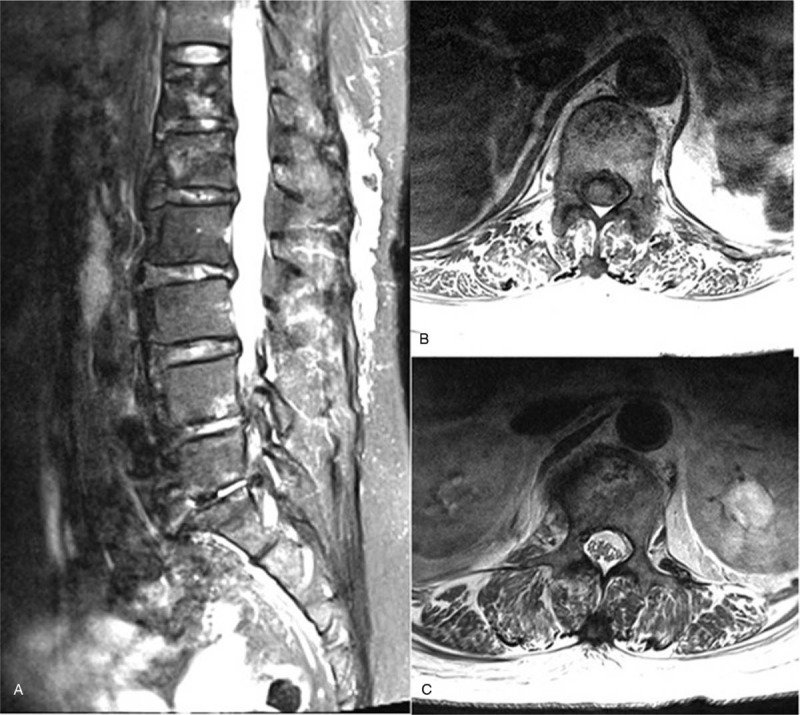
The enhanced T1 and T2 magnetic resonance imaging scan showing heterogenous signal intensity in the T12 (B) and L1 (C) vertebral bodies.

During the neurological examination, the patient complained of lumbar pain and pain in both buttocks, and tenderness on the mid back area. However, there were no neurological deficits. There were no specific findings on her x-ray except for a slight degenerative change. An abdominal and pelvic CT revealed intraosseous mottled air in the T12 and L1 vertebral bodies (Fig. 1). The enhanced T1 and T2 magnetic resonance imaging (MRI) scans showed heterogeneous signal intensity of vertebral bodies, suggestive of emphysematous osteomyelitis (Fig. 2). There was no definite epidural or paravertebral abscess.

Initial blood culture specimens obtained at the time of admission grew *E coli.* The patient was admitted to the Department of Infectious Medicine for management of sepsis and infective spondylitis. After 10 days of intravenous administration of vancomycin and cefepime, blood culture results were negative and CRP decreased from 9.88 to 2.55 mg/dL.

**Figure 3:**
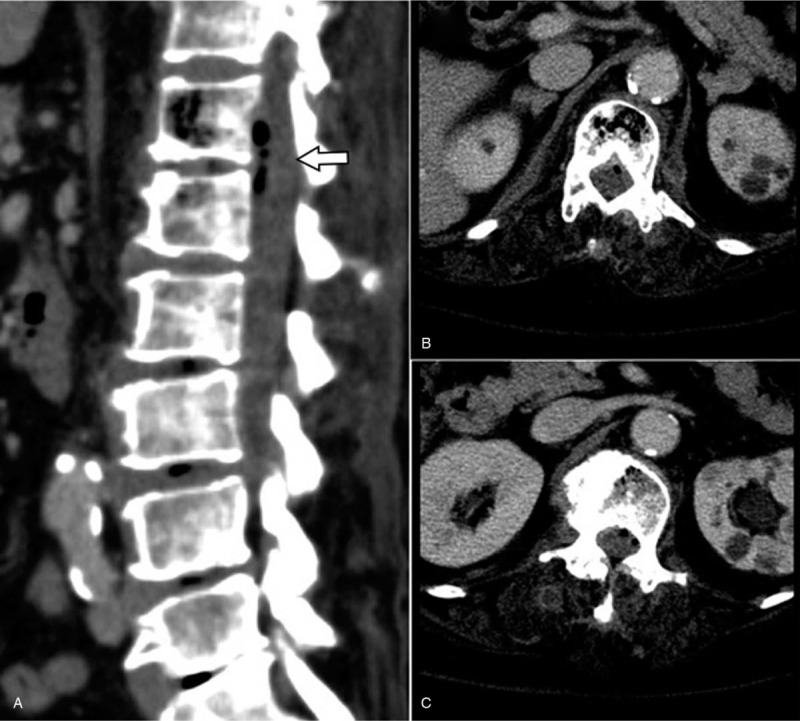
Follow-up computed tomography scan showing progressed emphysematous osteomyelitis at vertebral bodies of T12 (B) and L1, and newly developed air density at epidural space of T12-L1. (arrow) (A).

**Figure 4:**
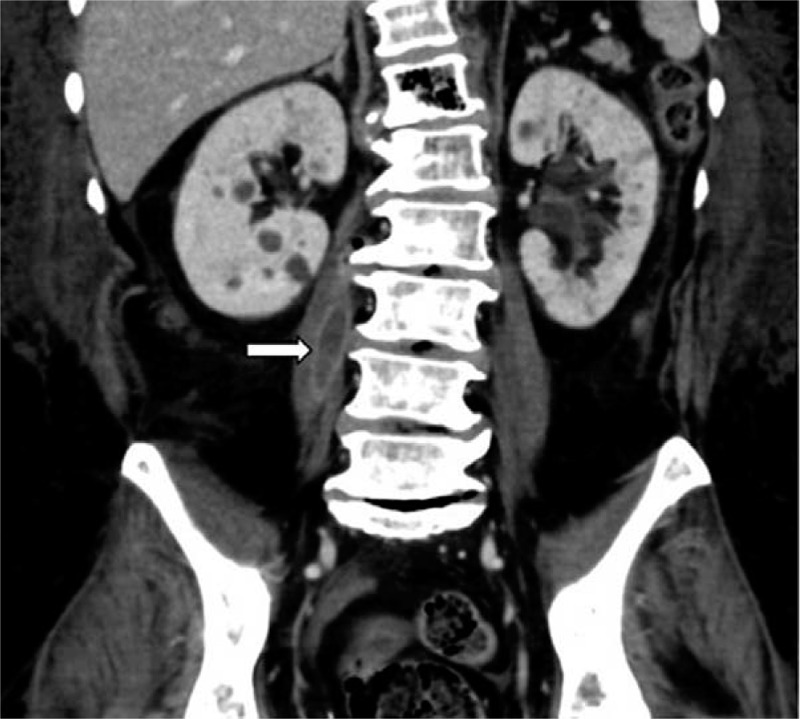
Follow-up computed tomography scan in coronal plane showing newly developed right psoas abscess at L1-L4 level. (arrow).

Although the laboratory results improved after antibiotic treatment, the patient continued to complain of aggravated back pain and inability to walk. A follow-up CT with contrast was performed, which revealed progressed emphysematous osteomyelitis at vertebral bodies of T12 and L1, and newly developed air density at the epidural space of the T12-L1 level with mild to moderate central canal compromise (Fig. 3). A right psoas abscess at L1-L4 level was also identified (Fig. 4).

**Figure 5:**
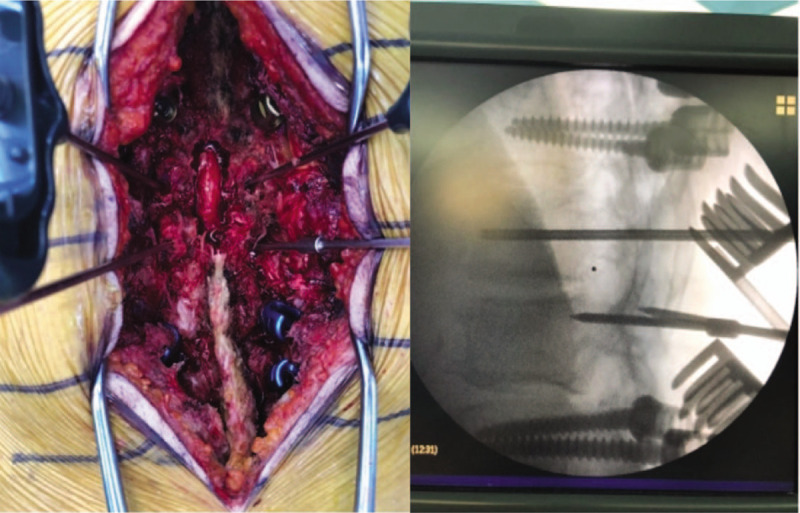
Transpedicular irrigation. After inserting the working cannula commonly used for cement injection into both pedicles, extensive irrigation was performed by injecting saline into one pedicle and suctioning it from the other side.

Surgery was performed to obtain specimens for histology and culture, identify culture strains, remove emphysematous lesions of the vertebral body and epidural space, and to reduce back pain by providing mechanical stability. During the posterior approach, pus was identified around the right T12-L1 facet joint. After laminectomy at the T12 to L1 levels, considerable amounts of pus were also identified under the ligamentum flavum. Pus-like thick fluid poured out from both T12 and L1 pedicles, and extensive transpedicular irrigation was done after collecting specimens from the vertebral body (Fig. 5). Posterior instrumentation with a pedicle screw was performed from T10 to L3, except T12, which was the most infected level. Posterolateral fusion with auto lamina bone graft was performed from T11 to L1.

**Figure 6:**
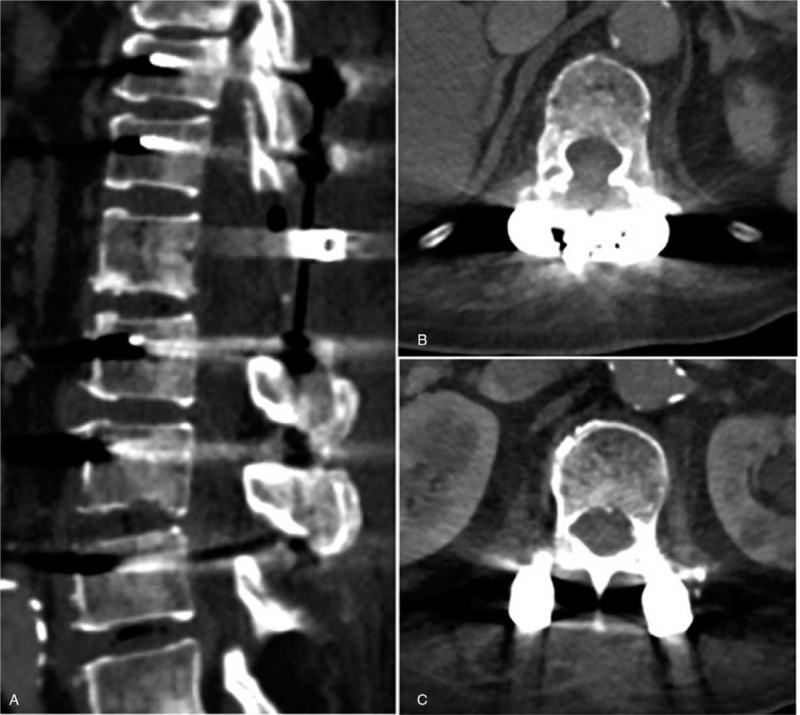
Postoperative computed tomography scan showing improved air density at epidural space (A) and significantly decreased air density at T12 (B) and L1 (C) vertebral bodies.

**Figure 7:**
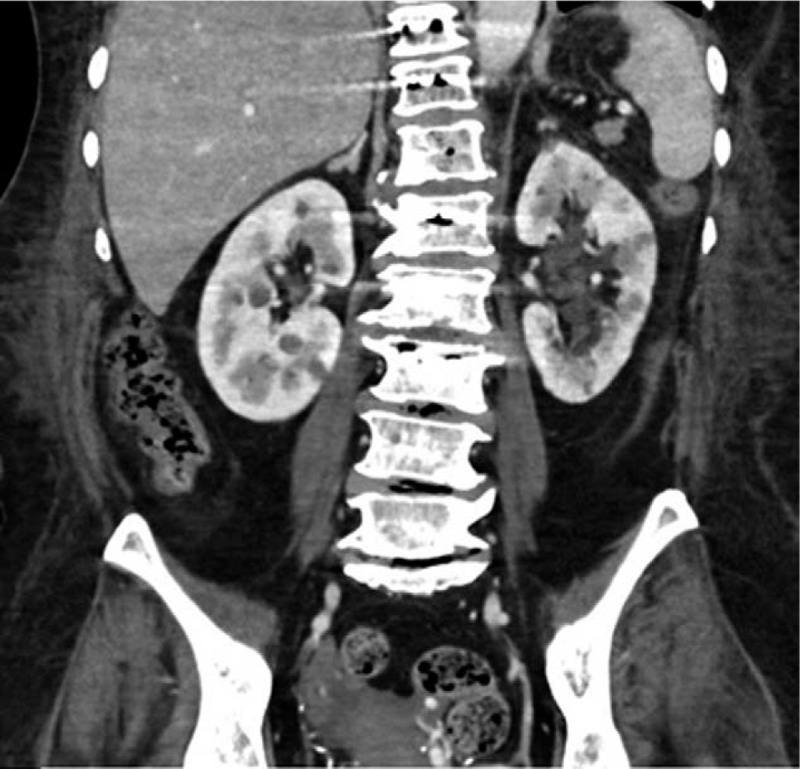
Last follow-up computed tomography scan in coronal plane showing improved right psoas abscess.

**Figure 8:**
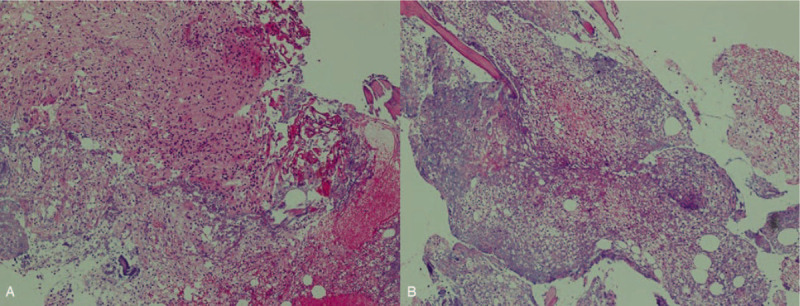
(A) Ghost cells without nucleus were widely distributed, which is suggestive of necrosis. (B) Many neutrophils were observed due to inflammatory reaction. (Hematoxylin and eosin stain, ×100).

*E coli* was identified in the surgical specimen, which was the same pathogen in the initial blood culture. Intravenous antibiotic therapy was continued as the bacteria were shown to be cefotaxime-sensitive. Postoperative vital signs were consistently stable and CRP decreased to 0.83 mg/dL, which is within the normal range, at postoperative day 7. The patient had a significant decrease in lower back pain after the surgery (visual analogue scale from 10 to 3) and had begun rehabilitation treatment for ambulation at 4 days post-surgery. After 20 days, the patient was able to walk using a walker. Intravenous antibiotics were continuously administered until 5 weeks postoperatively and she was then discharged with oral antibiotics. The final CT scan before discharge revealed significantly decreased air at T12 and L1 vertebral bodies and no air density in the epidural space (Fig. 6). The size of the right psoas abscess was also reduced compared to that on the preoperative CT (Fig. 7). The histopathology of the surgical specimens revealed ghost cells from necrosis and clustered neutrophils (Fig. 8).

## 3 Discussion

Emphysematous osteomyelitis is a rare and fatal disease. The main symptoms include fever and severe pain in the affected area. It is diagnosed by detecting intraosseous air on CT scans; however, air density, especially in axial skeleton, should be differentiated with degenerative change, osteonecrosis, or neoplasm. Emphysematous osteomyelitis may be more suspicious if fluid collection or abscess formation are detected in the adjacent tissue, or if intraosseous air shows an extensive and mottled pattern.^[6]^

A total of 45 cases have been reported since Ram et al^[1]^ first described three patients with emphysematous osteomyelitis in 1981. Luey et al^[7]^ analyzed 25 emphysematous osteomyelitis cases reported in the literature from 1981 to 2012. Since then, a relatively large number of cases (20 cases) have additionally been reported. We analyzed the characteristics of all of the cases reported so far.

There was no sex association, with 22 cases reported in female (48.9%), and the median age of patients was 56 years (range 14–79). DM, the most important known predisposing factor, was identified in 19 patients (42.2%), malignant tumor in 5 patients, and hematologic disorder in 5 patients. Uncontrolled DM is known to be highly associated with emphysematous osteomyelitis and high mortality^[8–10]^; however, in our patient, DM was relatively well controlled (Hb1A1C 6.8% [3.9–6.1]).

The most common site of infection was the vertebrae (n = 22), but infection has never been reported above the T5 level. Other locations included the pelvis (n = 11), femur (n = 11), tibia/fibula (n = 3), foot (n = 3), and sternum. (n = 2). Many of the reported cases, including our case, were monomicrobial (n = 33). The causative organisms were *E coli* (n = 14), *Klebsiella pneumoniae* (n = 10), *Bacteroides species* (n = 7), and *Fusobacterium* (n = 5), which are all gas-forming bacteria. Therefore, once intraosseous air density suggestive of emphysematous osteomyelitis is observed in imaging studies, empirical antibiotic therapy against *Enterobacteriaceae* family and anaerobes should be administered immediately. Because of the rarity of the disease, there is no consensus for effective antibiotic duration; therefore, antibiotics are usually administered for 4 to 6 weeks as recommended in routine osteomyelitis treatment.^[11]^

The main route of infection is known to be hematogenous spread in monomicrobial cases or contiguous spread from adjacent tissues in polymicrobial cases.^[7]^ The case presented here was a monomicrobial *E coli* infection, but it was unlikely to be a hematogenous spread as the patient developed the disease shortly after receiving injection therapy for her back. Surgical intervention should be considered when an abscess forms or when there is radiological evidence of progression.^[12]^ Among the reported 45 cases, 26 received surgical treatment and abscess formation in the adjacent tissues was observed in 24 cases. Surgery was performed in 20 cases of 24 patients with abscess formation, and the remaining 4 patients died during hospitalization. Surgical methods could be performed by removing pus using incision and drainage,^[5,7]^ or by debriding necrotic tissue extensively, followed by fusion and instrumentation if necessary.^[13,14]^ In this case, an abscess was also identified around the facet joint and spinal canal. Therefore, laminectomy and facetectomy were performed, and instrumentation and posterolateral fusion were carried out subsequently. Necrotic tissue inside the vertebral body was removed as much as possible, and then thorough irrigation was performed using the transpedicular technique.^[15]^

There were 14 patients who died during hospitalization with the mortality rate of 31.1%. *K pneumonia* infection had a particularly high mortality rate of 55.6%, with 5 of 9 patients dying during hospitalization. In addition, the majority of the deceased patients had vertebral infection (11 patients), and the mortality rate of vertebral infection was 50% (11/22 patients). The mortality rate of emphysematous osteomyelitis of vertebral infection is much higher than that of vertebral osteomyelitis, which has a mortality rate of 6% to 11%.^[16,17]^ It was also much higher than that of emphysematous osteomyelitis in any other location. The high mortality rate could be due to ambiguous presentation leading to delayed diagnosis or misdiagnosis, and difficulty treating with excision, unlike infections in extremities.

## 4 Conclusions

We reported a rare case of emphysematous osteomyelitis of the vertebral bodies due to *E coli* infection. Proper antibiotic therapy, early detection, and aggressive surgical intervention, where necessary, are all essential for the successful treatment of emphysematous osteomyelitis.

## Author contributions

**Conceptualization:** Byung Ho Lee, Yung Park, Joong Won Ha, Seong-Hwan Moon, Hwan-Mo Lee, Ji-Won Kwon.

**Data curation:** Sahyun Sung, Byung Ho Lee, Jung-Hwan Kim.

**Formal analysis:** Sahyun Sung.

**Investigation:** Sahyun Sung, Byung Ho Lee, Jung-Hwan Kim.

**Methodology:** Sahyun Sung, Seong-Hwan Moon, Hwan-Mo Lee, Ji-Won Kwon.

**Project administration:** Seong-Hwan Moon, Ji-Won Kwon.

**Resources:** Sahyun Sung, Byung Ho Lee, Jung-Hwan Kim.

**Supervision:** Sahyun Sung, Byung Ho Lee, Yung Park, Joong Won Ha, Seong-Hwan Moon, Hwan-Mo Lee, Ji-Won Kwon.

**Validation:** Yung Park, Joong Won Ha, Hwan-Mo Lee.

**Visualization:** Sahyun Sung, Ji-Won Kwon.

**Writing – original draft:** Sahyun Sung, Jung-Hwan Kim, Ji-Won Kwon.

**Writing – review & editing:** Sahyun Sung, Yung Park, Joong Won Ha, Ji-Won Kwon.
